# 1755. Interactive Provider Dashboards for Comparison of Outpatient Antimicrobial Prescribing for Respiratory and Otic Conditions in Urgent and Quick Care Clinics

**DOI:** 10.1093/ofid/ofac492.1385

**Published:** 2022-12-15

**Authors:** Kelly M Percival, Michael R de Blois, Patrick M Kinn, Lukasz Weiner, Dilek Ince

**Affiliations:** University of Iowa Hospitals & Clinics, Iowa City, Iowa; University of Iowa Hospitals & Clinics, Iowa City, Iowa; University of Iowa Hospitals & Clinics, Iowa City, Iowa; University of Iowa Hospitals & Clinics, Iowa City, Iowa; University of Iowa Hospitals & Clinics, Iowa City, Iowa

## Abstract

**Background:**

Due to antibiotic overutilization, development of antimicrobial stewardship (AMS) in the outpatient setting has become a necessary focus for AMS programs. The cornerstone of AMS is utilization of antimicrobial prescribing data to provide feedback to individual providers and compare providers to each other in order to promote improved prescribing practices. Respiratory infections, especially conditions that never require antimicrobials, are a common target for this feedback. We aimed to create interactive data visualization dashboards of end-users prescribing for these respiratory and otic conditions in order for AMS and urgent/quick care program staff to identify potential areas of intervention.

**Methods:**

A multidisciplinary work group consisting of data analysts, infectious diseases physicians, urgent/quick care physicians, and infectious diseases pharmacists determined dashboard content, layout, and comparison charts. Graph types were tested to determine optimal visual output. Using the electronic medical record ICD10 and antimicrobial prescription data was extracted for the encounter with focus on ICD10 codes for respiratory and otic conditions which never require antimicrobials. Rates were calculated as number of antimicrobial prescriptions over total number of visits for these conditions.

**Results:**

A multi-faceted dashboard was developed in Tableau® to view antimicrobial prescribing rates. Dashboard capabilities include the option to view individual provider prescribing rates compared to other de-identified providers, overall clinic prescribing rate, or individual rate compared to clinic rate. This allows for quick direct comparison of antimicrobial prescribing on the same graph by clinic to identify outliers (Figure 1) and allows visualization of trends in prescribing rates by clinics and providers over time (Figures 2,3).

Never Antimicrobial Prescribing Rate Comparison

**Figure:**
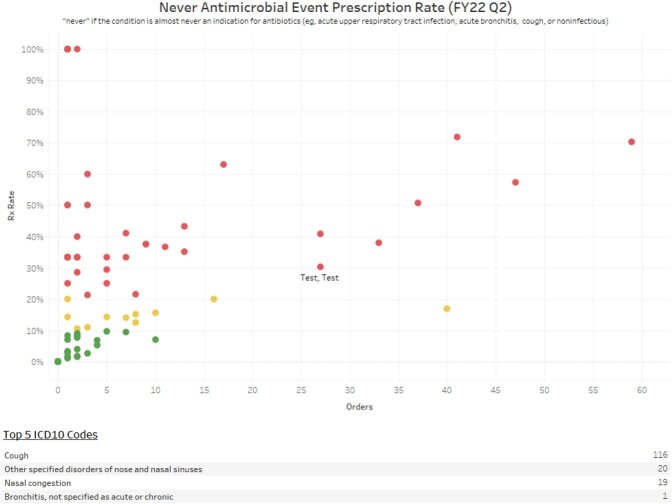

Scatter plot comparing individual prescribers rate of prescribing for never antimicrobial events

Clinic Antimicrobial Prescribing Rate Trend

**Figure:**
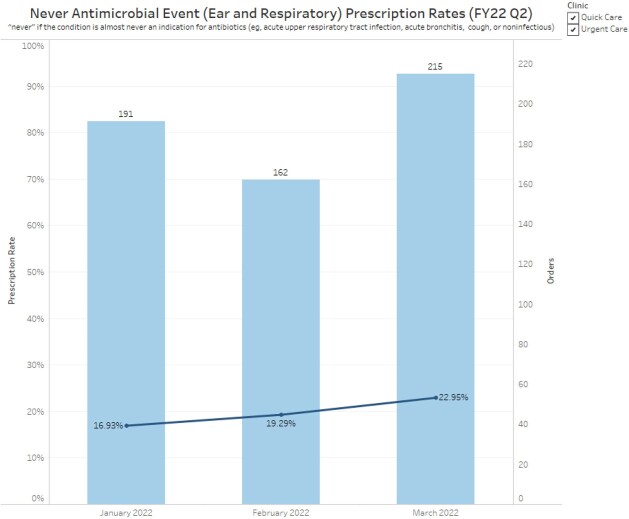

Clinic prescribing rate trend over time for never antimicrobial events

Prescriber and Clinic Antimicrobial Prescribing Rate Trend

**Figure:**
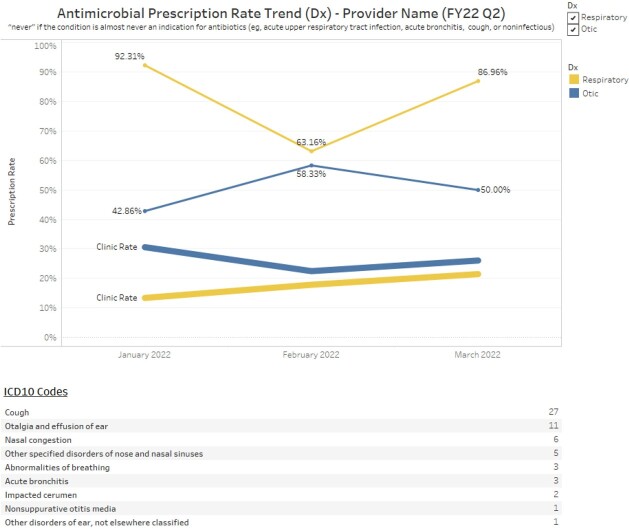

Individual prescriber rate compared to clinic rate over time for never antimicrobial events

**Conclusion:**

We developed an interactive outpatient AMS dashboard to visualize prescribing of antimicrobials for never respiratory and otic conditions. These dashboards can be used by providers along with AMS and clinic leadership to determine areas of intervention in the urgent/quick care setting.

**Disclosures:**

**Kelly M. Percival, PharmD**, Gilead Sciences Inc: Advisor/Consultant **Patrick M. Kinn, PharmD, MPH**, Gilead Sciences: Advisor/Consultant **Dilek Ince, MD**, Evergreen: Member of data monitoring board|Gilead Sciences: Grant/Research Support|Leidos: Grant/Research Support|Moderna: Grant/Research Support.

